# Supplementary material for: Mental Health and Health-Related Quality of Life of Children and Youth during the First Year of the COVID-19 Pandemic: Results from a Cross-Sectional Survey in Saskatchewan, Canada
Source: Children (Basel). 2023 Jun 3;10(6):1009. doi: 10.3390/children10061009 (PMC10297117; doi:10.3390/children10061009)
Supplement: Supplementary file 1 [file children-10-01009-s001.zip › children-2394485-supplementary.pdf]

Paper: Mental Health and Health-Related Quality of Life of Children and Youth during the First Year of the COVID-19 Pandemic: Results from a Cross-Sectional Survey in Saskatchewan, Canada
Nazeem Muhajarine et al. Children 2023, 10, 1009. https://doi.org/10.3390/children10061009

Supplemental file

Table S1: Bivariate Analysis of Anxiety, Depression, and Quality of Life in Children 8-18 Years During the First Year of the Pandemic in Saskatchewan, Canada.

Covariates	Anxiety		Depression		Quality of Life	
	Low (0-64)	Medium -High (>65)	Low (0-64)	Medium -High (>65)	High (71-100)	Low-Moderate (0-70)
Age						
	n=467		n=467		n=502	
8-11 years	208(92.0)	18(8.0)	210 (92.9)	16(7.1)	138(59.2)	95(40.8)
12-15 years	136(86.1)	22(13.9)	136(86.1)	22(13.9)	95(55.6)	76(44.4)
16-18 years	76(91.6)	7(8.4)	78(94.0)	5(6.0)	22(22.4)	76(77.6)
p-value	**0.139**		**0.040**		**<0.001**	
Grade						
	n=467		n=467		n=490	
Elementary (1-8)	287(90.8)	29(9.2)	289(91.5)	27(8.5)	203(61.7)	126(38.3)
High (9-12)	132(87.4)	19(12.6)	135(89.4)	16(10.6)	48(29.8)	113(70.2)
p-value	0.257		0.473		**<0.001**	
Gender						
	n=467		n=467		n=477	
Girl/Woman	190(88.4)	25(11.6)	193(89.9)	22(10.2)	111(50.5)	109(49.5)
Boy/Man	229(90.9)	23(9.1)	231(91.7)	21(8.3)	135(52.5)	122(47.5)
p-value	0.375		0.479		0.651	
Income						
	n=466		n=467		n=504	
<$100,000	146(87.4)	21(12.6)	149(89.2)	18(10.8)	101(55.5)	81(44.5)
[3]$100,000	180(96.8)	6(3.2)	180(96.8)	6(3.2)	106(52.7)	95(47.3)
Missing	93(82.3)	20(17.7)	95(83.3)	19(16.7)	49(40.5)	72(59.5)
p-value	**<0.001**		**<0.001**		**0.030**	
Ethnicity						
	n=421		n=421		n=451	
Ethnocultural minority	104(89.7)	12(10.3)	105(90.5)	11(9.5)	76(63.3)	44(36.7)
White	276(90.5)	29(9.5)	280(91.8)	25(8.2)	162(48.9)	169(51.1)
p-value	0.796		0.673		**0.007**	
Place of residence						
	n=415		n=415		n=443	

Mid-size city/town	150(94.3)	9(5.7)	150(94.3)	9(5.7)	85(52.1)	78(47.9)
Rural	67(88.2)	9(11.8)	71(93.4)	5(6.6)	44(53.0)	39(47.0)
Regina/Saskatoon	154(85.6)	26(14.4)	157(87.2)	23(12.8)	103(52.3)	94(47.7)
p-value	**0.030**		**0.053**		0.991	
Parental immigration status						
	n=430		n=430		n=467	
Both born in Canada	323(88.0)	44(12.0)	330(89.9)	37(10.1)	202(50.9)	195(49.1)
None/Either one born in Canada	61(96.8)	2(3.2)	62(98.4)	1(1.6)	38(54.3)	32(45.7)
p-value	**0.037**		**0.028**		0.599	
Household density						
	n=467		n=467		n=503	
£1 person/bedroom	192(85.7)	32(14.3)	192(85.7)	32(14.3)	118(47.8)	129(52.2)
>1 person/bedroom	227(93.4)	16(6.6)	232(95.5)	11(4.5)	138(53.9)	118(46.1)
p-value	**0.006**		**<0.001**		**0.169**	
Mental health before COVID						
	n=464		n=464		n=499	
Excellent	144(98.6)	2(1.4)	143(97.9)	3(2.1)	96(61.5)	60(38.5)
Very good/good	226(87.9)	31(12.1)	228(89.1)	28(10.9)	129(47.4)	143(52.6)
Fair/poor	47(77.0)	14(23.0)	50(80.6)	12(19.4)	27(38.0)	44(62.0)
p-value	**<0.001**		**<0.001**		**0.001**	
Schooling situation (Mode of learning)						
	n=438		n=437		n=471	
Either in-class or online	209(94.1)	13(5.9)	211(95.5)	10(4.5)	146(60.8)	94(39.2)
Both	189(87.5)	27(12.5)	189(87.5)	27(12.5)	81(35.1)	150(64.9)
p-value	**0.016**		**0.003**		**<0.001**	
COVID cases in classroom						
	n=467		n=467		n=502	
Yes	133(93.7)	9(6.3)	135(95.1)	7(4.9)	77(50.3)	76(49.7)
No	155(92.8)	12(7.2)	153(91.6)	14(8.4)	99(56.3)	77(43.8)
Missing	131(82.9)	27(17.1)	136(86.1)	22(13.9)	79(45.7)	94(54.3)
p-value	**0.002**		**0.024**		**0.140**	
Household conflicts						

	n=467		n=467		n=503	
Lot/somewhat more	95(78.5)	26(21.5)	94(77.7)	27(22.3)	38(29.0)	93(71.0)
No real change	191(95.0)	10(5.0)	192(95.5)	9(4.5)	131(61.5)	82(38.5)
Lot/somewhat less	29(96.7)	1(3.3)	29(96.7)	1(3.3)	21(70.0)	9(30.0)
Varied	105(91.3)	10(8.7)	109(94.8)	6(5.2)	66(51.2)	63(48.8)
p-value	**<0.001**		**<0.001**		**<0.001**	
Sleep						
	n=442		n=443		n=477	
<8 hours	86(89.6)	10(10.4)	83(86.5)	13(13.5)	52(46.8)	59(53.2)
^{3}8 hours	318(91.9)	28(8.1)	323(93.1)	24(6.9)	193(52.7)	173(47.3)
p-value	0.472		**0.038**		0.277	
Physical activity						
	n=467		n=466		n=501	
7 days/week MVPA	72(88.9)	9(11.1)	75(92.6)	6(7.4)	56(60.9)	36(39.1)
<7 days/week MVPA	347(89.9)	39(10.1)	348(90.4)	37(9.6)	199(48.7)	210(51.3)
p-value	0.786		0.533		**0.034**	
Change in physical activity						
	n=466		n=465		n=502	
Lot/little less active	281(91.5)	26(8.5)	283(92.5)	23(7.5)	154(47.1)	173(52.9)
About the same	72(93.5)	5(6.5)	74(96.1)	3(3.9)	54(63.5)	31(36.5)
Little/lot more active	65(79.3)	17(20.7)	66(80.5)	16(19.5)	47(52.2)	43(47.8)
p-value	**0.003**		**<0.001**		**0.025**	
Screen time						
	n=408		n=408		n=443	
<3 hours	144(95.4)	7(4.6)	148(98.0)	3(2.0)	107(66.5)	54(33.5)
^{3}3 hours	226(87.9)	31(12.1)	225(87.5)	32(12.5)	118(42.1)	162(57.9)
p-value	**0.013**		**<0.001**		**<0.001**	

Table S2: Full Regression Model for Factors Associated with Medium-High Anxiety.

	Medium-high Anxiety	
	OR (95%CI)	*p-value*
Main effects		
Age group (years)		

8-11	4.54 (1.33-15.47)	**0.016**
16-18	1.17 (0.28-4.89)	0.833
12-15	Reference	
Gender		
Girl/Woman	2.07 (0.74-5.81)	0.165
Boy/Man	Reference	
Income		
<$100,000	1.20 (0.37-3.93)	0.765
Missing	13.50 (4.13-44.15)	**<0.001**
≥$100,000	Reference	
Ethnicity		
Ethnocultural minority	43.25 (4.18-447.81)	**0.002**
White	Reference	
Place of residence		
Rural	8.44 (2.04-34.89)	**0.003**
Regina/Saskatoon	4.07 (1.07-15.46)	**0.039**
Mid-size city/towns	Reference	
Household density		
>1 person/bedroom	0.13 (0.04-0.42)	**0.001**
£1 person/bedroom	Reference	
Mental health before COVID		
Very good/good	6.18 (1.11-34.36)	**0.038**
Fair/poor	18.49 (2.69-127.03)	**0.003**
Excellent	Reference	
Schooling situation (Mode of learning)		
Either in-class or online	1.19 (0.48-2.92)	0.712
Both	Reference	
Screen time		
^{3}3 hours	3.07 (1.16-8.12)	**0.023**
<3 hours	Reference	
	Interactions	
Age X Ethnicity		
8-11 years X Ethnocultural minority	0.04 (0.003-0.51)	**0.013**
16-18 years X Ethnocultural minority	0.04 (0.003-0.57)	**0.017**
Gender X Ethnicity		

Girl/Woman X Ethnocultural minority	0.03 (0.003-0.26)	**0.002**

Note: Odds Ratio. Parsimonious model shown in table; adjusted for age, gender, income, ethnicity, parental immigration status, place of residence, household density, mental health before COVID, schooling situation, COVID cases in classroom, household tension/conflict, change in physical activity and screen time, age X ethnicity, gender X ethnicity, income X ethnicity, age X parental immigration status, gender X parental immigration status, and income X parental immigration status. Model fit statistics: AIC=135.557, BIC=200.142

Table S3: Full Regression Model for Factors Associated with Medium-High Depression.

	Medium-high Depression	
	OR (95%CI)	*p-value*
Main effects		
Age group (years)		
8-11	3.48 (1.03-11.76)	**0.044**
16-18	0.19 (0.04-0.89)	**0.034**
12-15	Reference	
Gender		
Girl/Woman	1.61 (0.52-4.98)	0.408
Boy/Man	Reference	
Income		
<$100,000	1.07 (0.34-3.30)	0.912
Missing	2.25 (0.52 -9.66)	0.276
³$100,000	Reference	
Ethnicity		
Ethnocultural minority	0.89 (0.22-3.56)	0.869
White	Reference	
Place of residence		
Rural	6.60 (1.01-43.17)	**0.049**
Regina/Saskatoon	6.18 (1.45-26.29)	**0.014**
Mid-size city/towns	Reference	
Household density		

>1 person/bedroom	0.06 (0.01-0.24)	**<0.001**
£1 person/bedroom	Reference	
Mental health before COVID		
Very good/good	0.69 (0.16-2.89)	0.609
Fair/poor	3.94 (0.68-22.84)	0.126
Excellent	Reference	
Household conflicts		
Lot/somewhat more	10.84 (2.28-51.51)	**0.003**
Lot/somewhat less	3.15 (0.35-27.97)	0.304
Varied	5.58 (1-31.23)	0.051
No real change	Reference	
Sleep		
^{3}8 hours	0.07 (0.02-0.26)	**<0.001**
<8 hours	Reference	
Screen time		
^{3}3 hours	5.2 (1.52-17.79)	**0.009**
<3 hours	Reference	

Note: Odds Ratio. Parsimonious model shown in table; adjusted for age, gender, income, ethnicity, parental immigration status, place of residence, household density, mental health before COVID, schooling situation, COVID cases in classroom, household tension/conflict, sleep, change in physical activity and screen time, age X ethnicity, gender X ethnicity, income X ethnicity, age X parental immigration status, gender X parental immigration status, and income X parental immigration status. Model fit statistics: AIC=111.963, BIC=176.183

Table S4: Full Regression Model for Factors Associated with Low-Moderate Quality of Life**.**

	Low-moderate Quality of Life	
	OR (95%CI)	*p-value*
Main effects		
Age group (years)		
8-11	0.89 (0.36-2.21)	0.808
16-18	5.88 (1.66-20.86)	**0.006**

12-15	Reference	
Gender		
Girl/Woman	1.37(0.69-2.74)	0.37
Boy/Man	Reference	
Income		
<$100,000	0.82 (0.30-2.38)	0.707
Missing	2.77 (0.75-10.24)	0.127
≥$100,000	Reference	
Ethnicity		
Ethnocultural minority	0.89 (0.18-4.32)	0.883
White	Reference	
Place of residence		
Rural	0.78 (0.25-2.39)	0.659
Regina/Saskatoon	0.56 (0.25-1.26)	0.161
Mid-size city/towns	Reference	
Parental immigration status		
None/Either one born in Canada	0.29 (0.74-1.13)	0.075
Both born in Canada	Reference	
Mental health before COVID		
Very good/good	3.25 (1.56-6.79)	**0.002**
Fair/poor	2.51 (0.70-9)	0.157
Excellent	Reference	
Schooling situation (Mode of learning)		
Either in-class or online	0.43 (0.165-1.10)	0.078
Both	Reference	
COVID cases in classroom		
Yes	1.01 (0.41-2.47)	0.987
Missing	2.85 (1.20-6.78)	**0.018**
No	Reference	
Household conflicts		
Lot/somewhat more	3.38 (1.46-7.85)	**0.005**
Lot/somewhat less	0.43 (0.13-1.46)	0.178
Varied	1.29 (0.50-3.35)	0.598
No real change	Reference	
Sleep		
^{3}8 hours	0.79 (0.31-2.06)	0.635

< 8 hours	Reference	
Change in physical activity		
Lot/little less active	3.83 (1.46-10.04)	**0.006**
Little/lot more active	2.01 (0.62-6.50)	0.241
About the same	Reference	
Screen time		
^{3}3 hours	2.70 (1.11-6.58)	**0.029**
<3 hours	Reference	
	Interactions	
Age X Ethnicity		
8-11 years X Ethnocultural minority	0.59 (0.008-0.42)	**0.005**
16-18 years X Ethnocultural minority	1.08 (0.15-7.98)	0.943
Income X Ethnicity		
<$100,000 X Ethnocultural minority	8.30 (1.18-58.55)	**0.034**
Missing X Ethnocultural minority	2.44 (0.23-25.90)	0.459
Age X Parental immigration status		
8-11 years X None/Either one born in Canada	10.55 (1.35-82.22)	**0.025**
16-18 years X None/Either one born in Canada	0.60 (0.61-5.97)	0.666

Note: Odds Ratio. Parsimonious model shown in table; adjusted for age, gender, income, ethnicity, parental immigration status, place of residence, household density, mental health before COVID, schooling situation, COVID cases in classroom, household tension/conflict, sleep, physical activity, change in physical activity and screen time, age X ethnicity, gender X ethnicity, income X ethnicity, age X parental immigration status, gender X parental immigration status, and income X parental immigration status.

Model fit statistics: AIC=330.841, BIC=436.441

Paper: Mental Health and Health-Related Quality of Life of Children and Youth during the First Year of the COVID-19 Pandemic: Results from a Cross-Sectional Survey in Saskatchewan, Canada
Nazeem Muhajarine et al. Children 2023, 10, 1009. https://doi.org/10.3390/children10061009

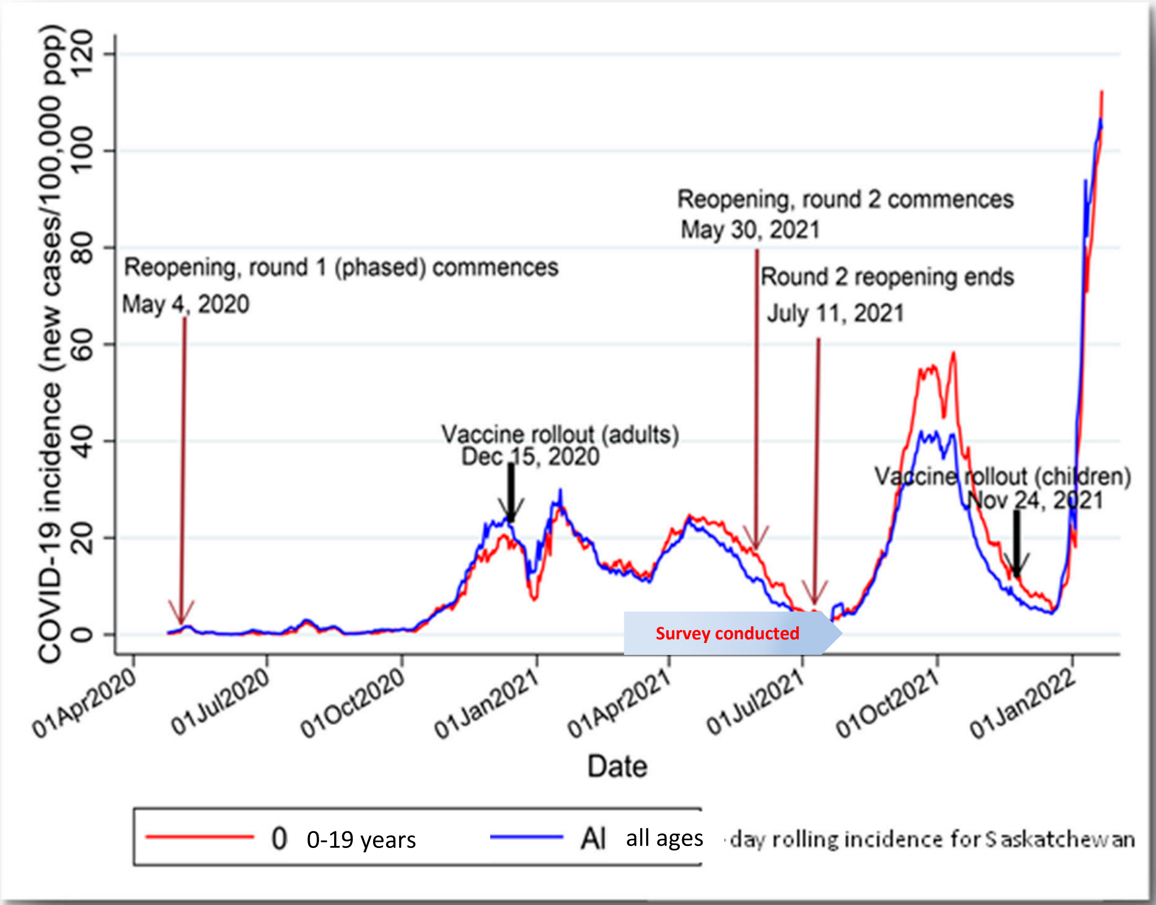

Figure S1: Incidence of COVID-19 in Saskatchewan, Canada.

Paper: Mental Health and Health-Related Quality of Life of Children and Youth during the First Year of the COVID-19 Pandemic: Results from a Cross-Sectional Survey in Saskatchewan, Canada
Nazeem Muhajarine et al. Children 2023, 10, 1009. https://doi.org/10.3390/children10061009

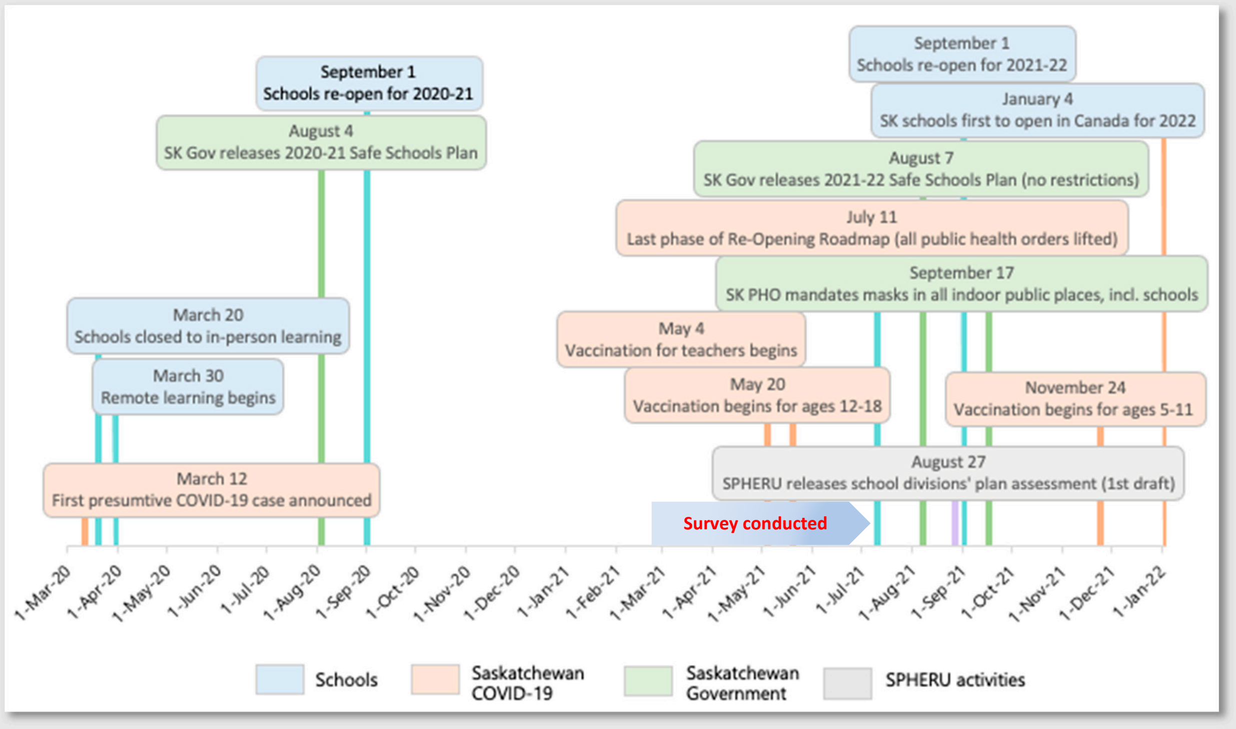

Figure S2: Timeline of COVID-19 and Elementary and Secondary Schools in Saskatchewan, Canada**.**
